# Supplementary figures and images for: HIV-1 accessory protein Vpr possesses a cryptic p300-dependent transcription-promoting activity that is blocked by histone deacetylases in CD4+ T cells
Source: PLoS Pathog. 2025 Sep 3;21(9):e1013073. doi: 10.1371/journal.ppat.1013073 (PMC12419630; doi:10.1371/journal.ppat.1013073)

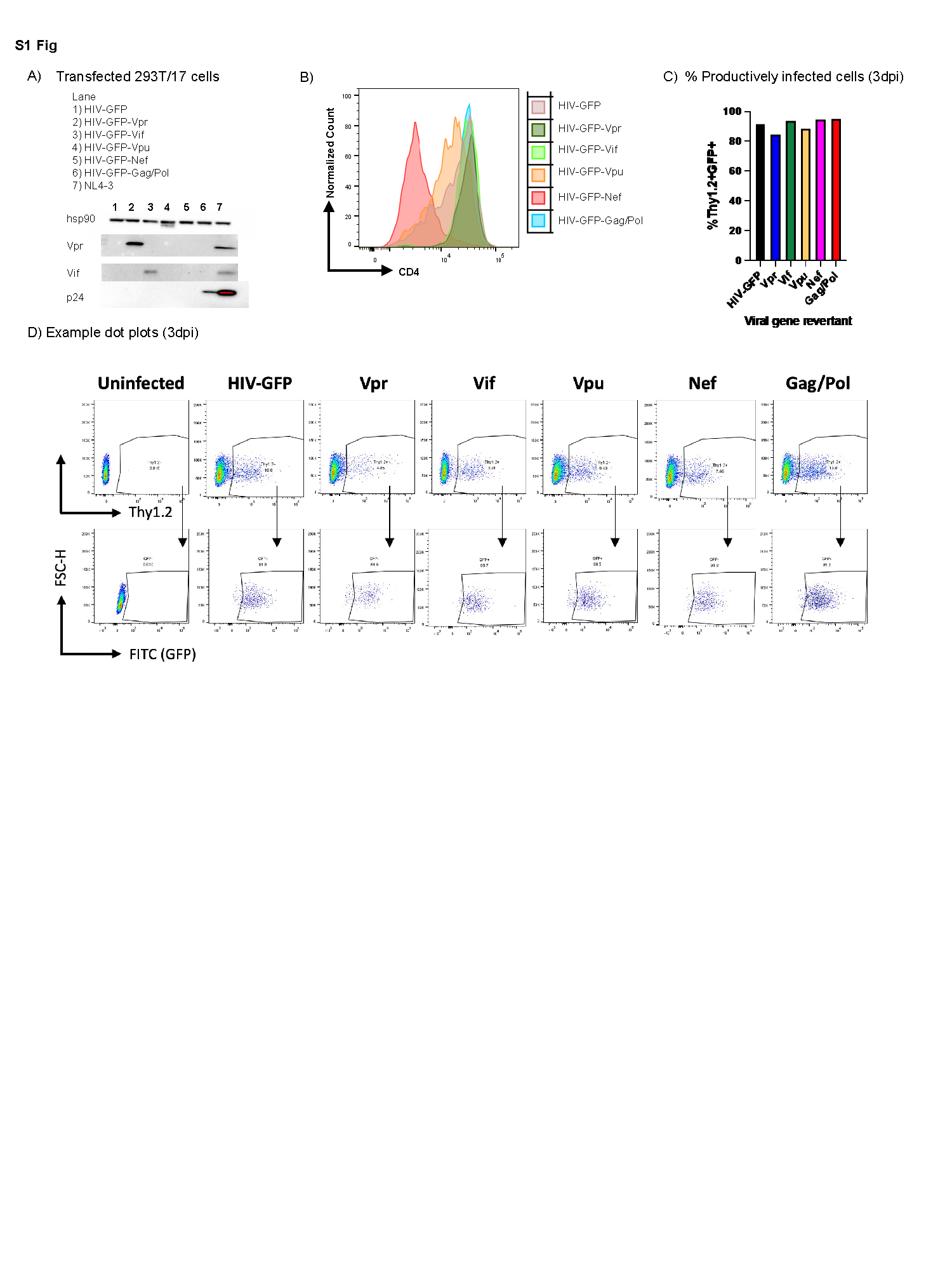

Supplement: S1 Fig — A) Western blot for Vif, Vpr, and p24 of 293T cells transfected with HIV-GFP revertant plasmids. B) CD4 expression in CD4+ T cells infected with HIV-GFP revertant viruses. C) Quantification of GFP+ CD4+ T cells within Thy1.2+ population for each virus. D) Representative flow plots for infection with each revertant virus. GFP+ cells were gated within Thy1.2+ cells. (TIF) [file ppat.1013073.s001.tif]

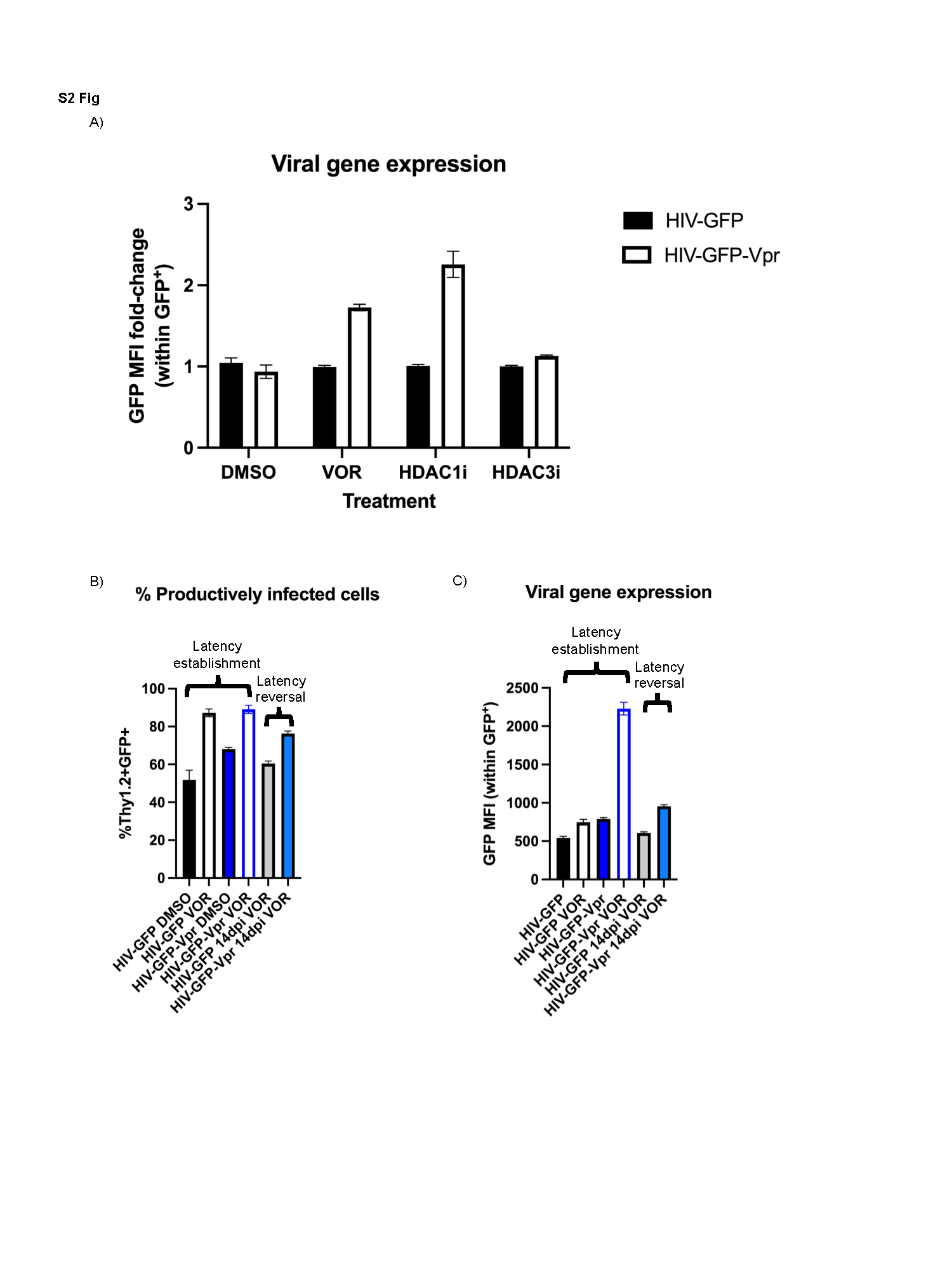

Supplement: S2 Fig — A) GFP median fluorescence intensity (MFI) within GFP+ cells in HIV-GFP- or HIV-GFP-Vpr-infected cells treated with histone deacetylase inhibitor (HDACi) for 14 days. Results are normalized to GFP MFI within HIV-GFP-infected cells for each condition. B-C) Latency reversal versus latency prevention: Cells infected with HIV-GFP or HIV-GFP-Vpr were cultured for 14 days as in A). After 14 days (14dpi), DMSO-treated cells were treated with VOR (vorinostat) for 24h before B) %GFP+ cells and C) GFP MFI within GFP+ cells was assessed by flow cytometry. (TIF) [file ppat.1013073.s002.tif]

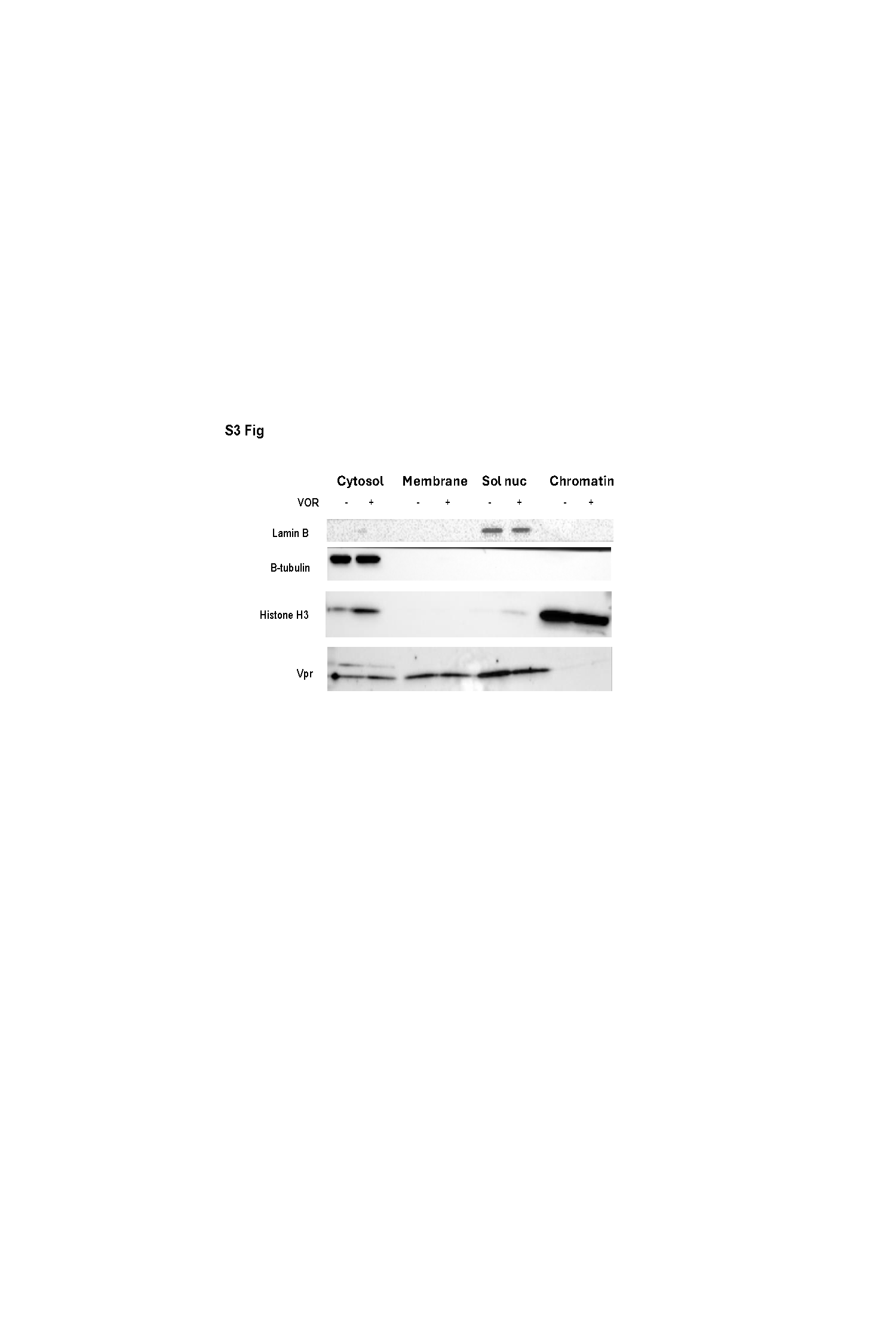

Supplement: S3 Fig — Western blot with indicated antibody on cell fractions from cells treated with DMSO or vorinostat. VOR = vorinostat. Sol nuc = soluble nuclear fraction. (TIF) [file ppat.1013073.s003.tif]

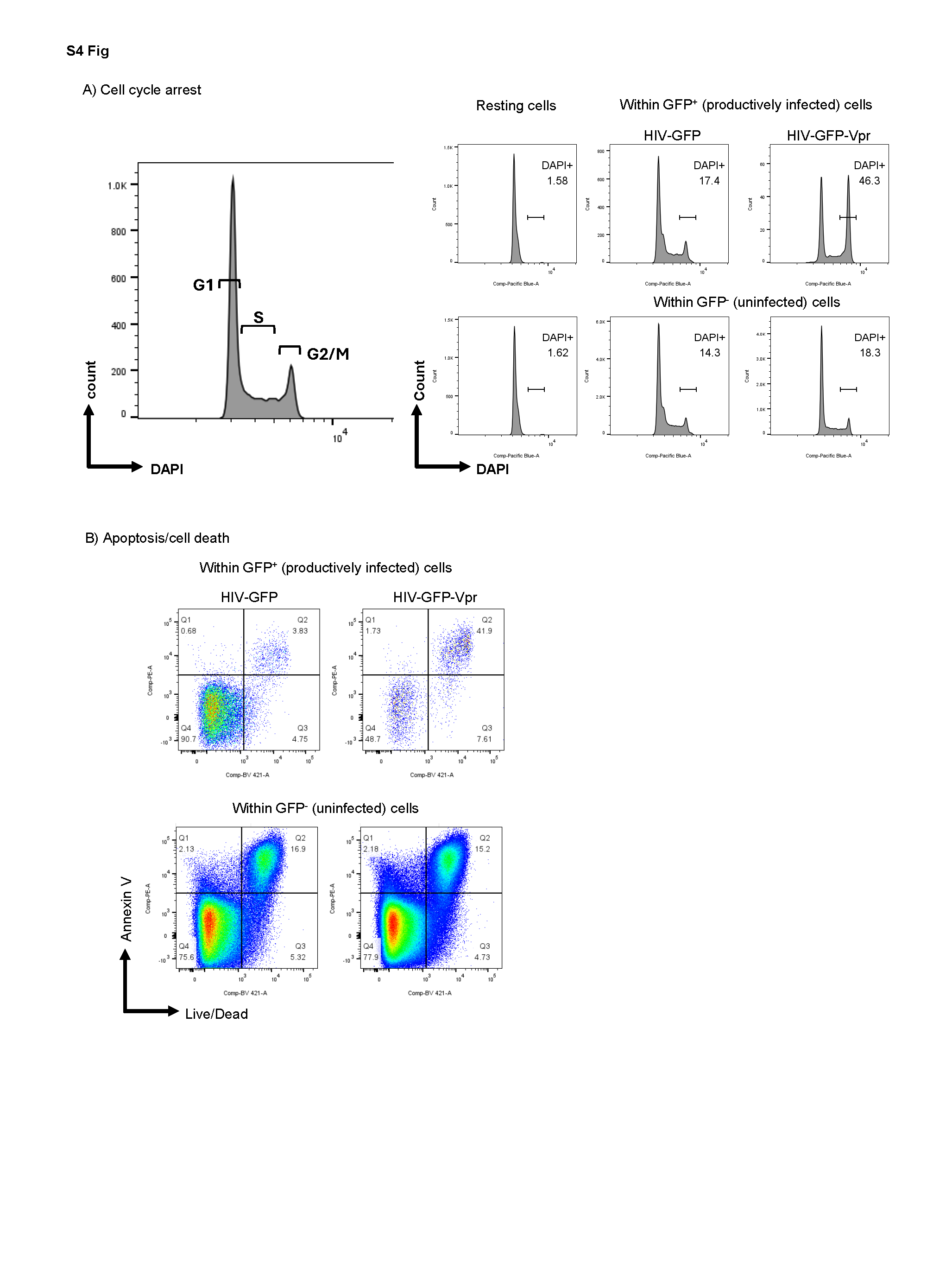

Supplement: S4 Fig — A) Left: example histogram of DAPI staining for cell cycle. Right: % cells in G2/M in each condition 7 days post-infection. B) Representative flow plots of cells stained with Annexin V and Zombie Violet to measure apoptosis and cell death 2 days post-infection. (TIF) [file ppat.1013073.s004.tif]

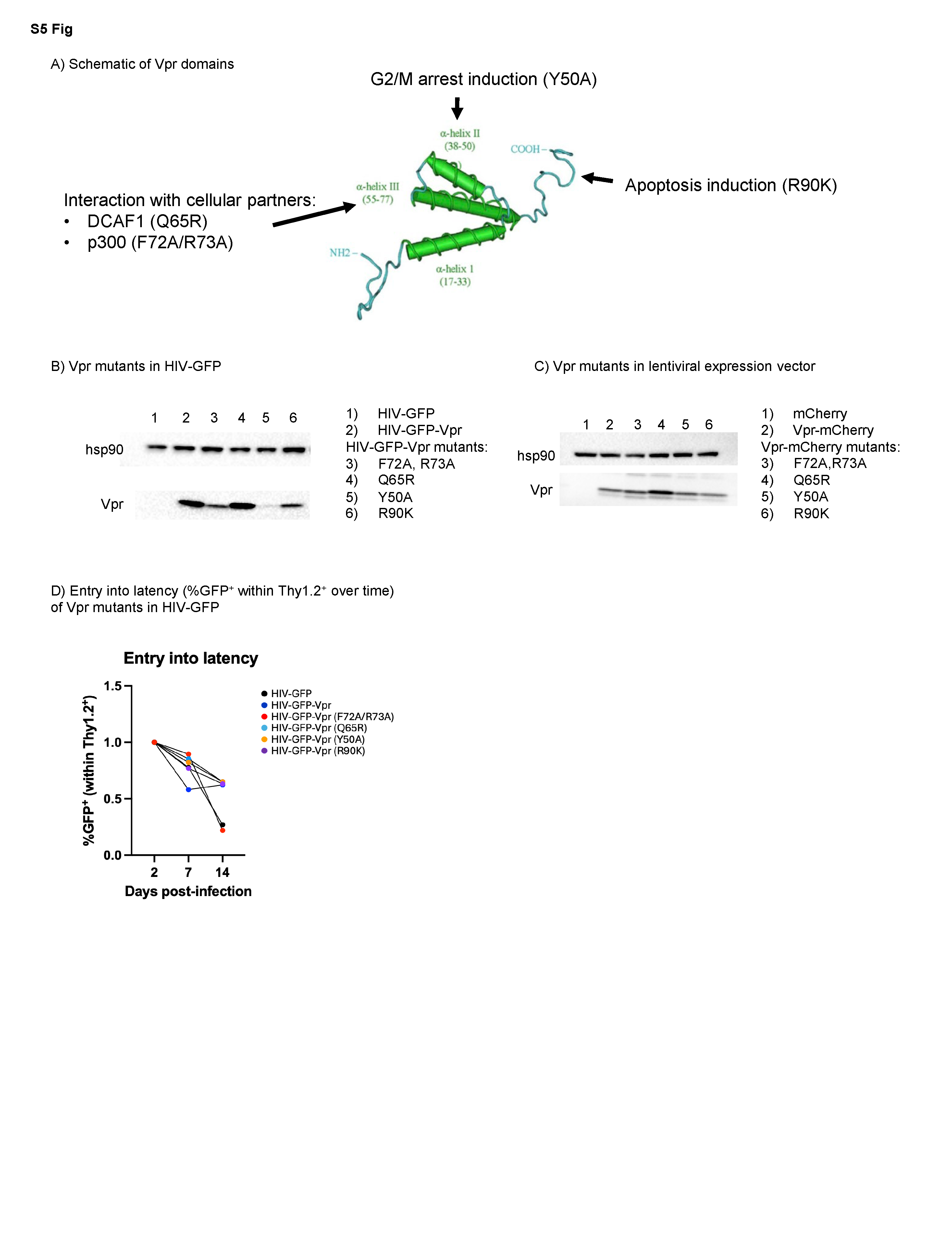

Supplement: S5 Fig — A) Schematic of Vpr domains adapted from Guenzel et al., 2014 [87]. Induction of cell cycle arrest and apoptosis/cell death by Vpr functional mutant viruses. Vpr expression in 293T cells transfected with mutant B) HIV-GFP-Vpr and C) Vpr-mCherry plasmids. (TIF) [file ppat.1013073.s005.tif]

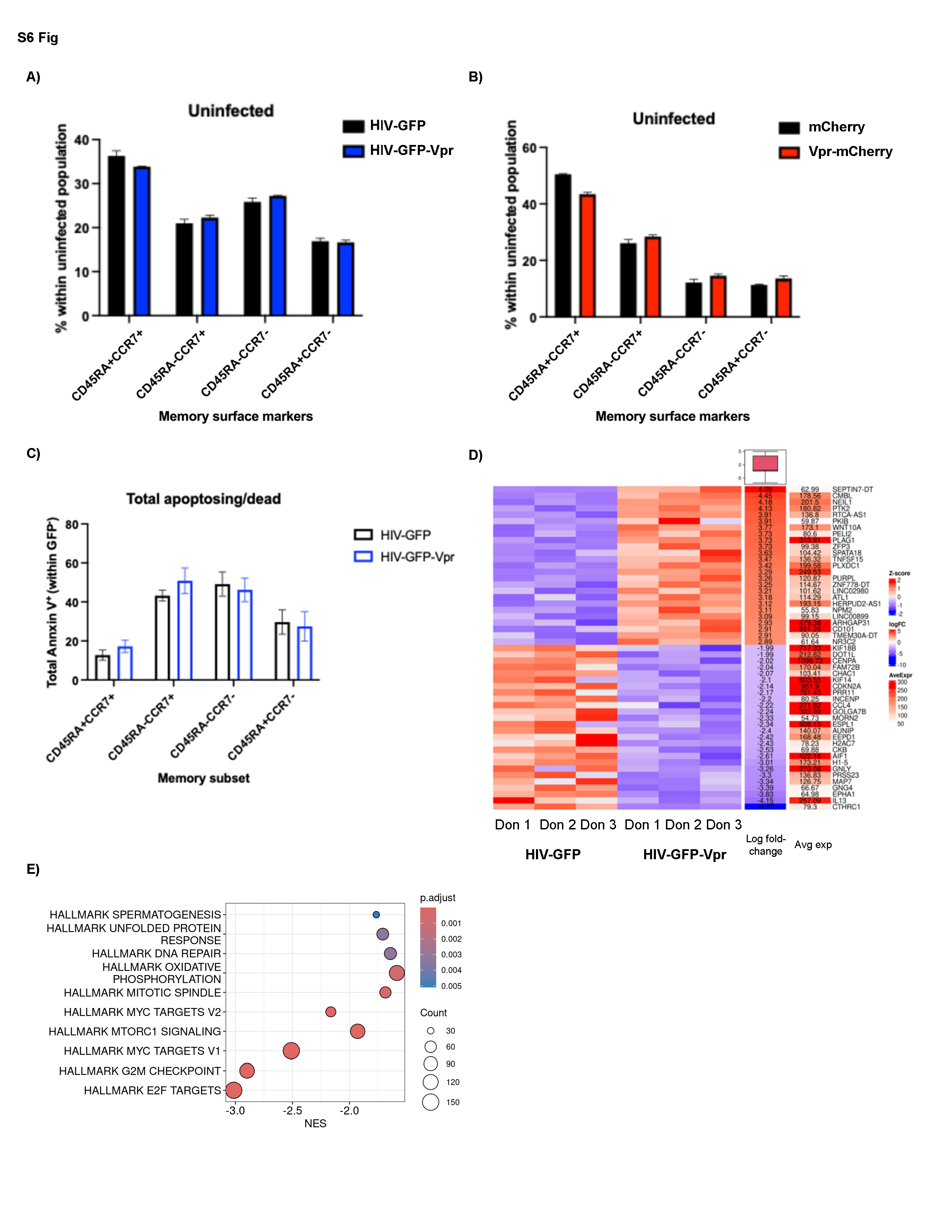

Supplement: S6 Fig — Representative data for %GFP- (uninfected) cells in each subset as defined by CCR7 and CD45RA in samples infected with A) HIV-GFP-Vpr or HIV-GFP or B) Vpr-mCherry or mCherry lentivirus. C) Representative plot of %Annexin V cells in each subset from cells infected with HIV-GFP-Vpr or HIV-GFP. D) Heat map of differentially expressed genes (DEGs) in GFP+ CD4+ T cells infected with HIV-GFP-Vpr or HIV-GFP (n = 3). E) gene-set enrichment analysis for all DEGs. (TIF) [file ppat.1013073.s006.tif]
