# Supplementary material for: HIV-1 accessory protein Vpr possesses a cryptic p300-dependent transcription-promoting activity that is blocked by histone deacetylases in CD4+ T cells
Source: PLoS Pathog. 2025 Sep 3;21(9):e1013073. doi: 10.1371/journal.ppat.1013073 (PMC12419630; doi:10.1371/journal.ppat.1013073)
Supplement: Attachment — Submitted filename: reponse to reviewers_Final.docx [file ppat.1013073.s007.docx]

**Response to reviewers**

**Reviewers' Comments:**

Reviewer's Responses to Questions

**Part I - Summary**
Please use this section to discuss strengths/weaknesses of study, novelty/significance, general execution and scholarship.

Reviewer #1: Lewis et al. assess the role of HIV-1 Vpr and HDAC inhibition in latent reservoir formation and HIV-1 promoter activity. They tested a series of proviral constructs in primary CD4+ T cells and determined that Vpr promotes LTR activity in the presence of the HDAC inhibitor Vorinostat. Further examination of this phenomenon suggested that Vpr promotes a central memory phenotype and that these cells are more resistant to Vpr-induced apoptosis. While several of the experimental observations herein confirm previous observations from prior studies, the novelty comes from the extensive use of primary CD4+ T cells. I have some concerns regarding the interpretation of some of the results and the magnitude of the changes induced by Vpr. Questions and concerns are detailed below.

Reviewer #2: Lewis et al explores activities of HIV-1 Vpr in CD4+ T cells with a focus of on whether Vpr influences HIV-1 transcription and the establishment of latency. Key findings are that Vpr and HDACs may interact to repress HIV transcription, and this activity is dependent on p300. In addition, Vpr influences primary CD4+ T cell survival and an expansion of Tcm cells, which have been shown to harbor latent proviruses. Strengths of the paper are the general approach of utilizing primary CD4+ T cells, the use of HIV-1 clones with a single accessory protein and utilizing Vpr mutations to map domains to specific functions that suggest Vpr may be through multiple mechanisms influencing HIV replication in primary CD4+ T cells. However, the potential multiple activities of Vpr, clouds the primary message of the paper which tries to emphasize the Vpr-HDAC-p300 axis. There are also some concerns as to the modest data and lack of exploring and discussing direct and indirect mechanisms of Vpr and HIV transcription. Specific comments are below.

1) The data in which Vpr+Vorinstat, a HDACi, examines how HDACs might alter HIV-1 infection, transcription, and latency show about a two-fold difference in HIV-1 expression, as monitored by GFP MFI and HIV-1 transcription. There is no discussion whether these differences are significant.

**We thank the reviewer for recommending that we be clearer in this respect. We performed Wilcoxon matched-pairs signed-rank test on the fold-changes of the GFP MFIs for the combined donors when n**$\boldsymbol{\geq}$**6 as described in the methods section, and we annotated the graphs in the supplemental figures to indicate significance. We originally chose to place these ‘combined donor’ graphs in the supplemental figures and one representative graph in the main figures, but based on the reviewer’s suggestion, we have now moved the graphs with the combined donors and statistical analysis to the main figures.**

2) It is difficult to know if Vpr+Vor reflects difference in transitioning to latency since no experiments are performed to look at the ability to reactivate proviruses after the extended culturing.

**We thank the reviewer for their suggestion. We have now included an experiment in which HIV-GFP or HIV-GFP-Vpr-infected cells are treated with vorinostat 2 weeks after infection (after the virus has gone into latency) to assess differences in ability to reactivate latent virus (Fig S2C,D). Interestingly, we observed that the combination of Vpr and vorinistat was less apparent in this latency reversal situation compared to Vpr expression from the outset of infection. We speculate that this is due to the lower level of Vpr expression when the virus has already entered latency, as well as the lower activity of HDACs in resting CD4 T cells. Thus, we speculate that this combined effect is specific to the onset of latency.**

3) It is stated that the percentage or number of cells that are expressing HIV do not significantly change, however, the flow cytometry profiles for the different experiments and different viruses suggest a great deal of variation between the different viruses and experiments. Does this reflect different fitness between viruses, experimental variation, or some other variable?

**Each experiment involves infection of cells from a different donor, so the variability reflects the heterogeneity across human populations. Median fluorescence intensity can vary by day, simply as a result of technical variability generated by the flow cytometer. For this reason, we chose to perform statistical analysis for MFI on the conditions within a single time point, rather than over time. Finally, the variation in infection levels with the individual revertant viruses we believe to be due to the functions of the different proteins, each of which play a role in viral life cycle and some of which, like Vpr, are cytotoxic. We have added some text to the manuscript to clarify this point.**

4) The use of different HDAC inhibitors would be interesting in that it might address potential off-targets of Vor, and provide insights into the role of different classes of HDACs.
**We thank the reviewer for this suggestion. We have now performed this experiment and included the results in Fig. S2. The results suggest that HDAC1 and/or HDAC2 may be the critical HDAC responsible for counteracting the pro-transcriptional activity of Vpr.**

5) The data with the trans expression of Vpr seemed modest and at times contradictory to the data obtained with the viruses. Are these significant differences?

**The results with trans Vpr show the same trends as the cis Vpr experiments. For GFP MFI, there were statistically significant differences between Vpr-mCherry lentivirus-transduced cells treated with vorinostat compared to mCherry lentivirus-infected cells on weeks 1 and 2, as well as several statistically significant differences on week one. This statistical analysis is now included in Figure 2D. The observed trend is the same as for cis Vpr expression, but because of the small percentage of co-infected cells remaining by week three, we only analyzed data for two donors for week three in these experiments, making inappropriate to perform statistical analyses. We again observed the same trends for the different Vpr mutants in trans, although we also saw an abrogation of the combined effect of Vpr and vorinostat in the DCAF1-binding mutant in addition to the p300-binding mutant. We suspect this may be due to overexpression of this mutant when expressed in trans. Finally, we have now performed memory subset composition analysis in one additional donor following trans Vpr expression so that we would have sufficiently large n to perform statistical analysis (n=6; Figure 5B). Although the effect is not statistically significant (p=0.0625), we observed the same trend in 5 out of 6 donors.**

6) The impact of Vpr on Tcm is interesting. It would be informative if they examined if this could lead to more latently infected cells by trying to reverse latency.
**We agree with the reviewer that this in an interesting finding. We also agree that there are several experiments that could be carried out to follow up on the significance of this finding. Previous studies have shown that the long-lived resting TCM compartment is an important latent HIV-1 reservoir (Song et al., 2020; reviewed in Lee and Lichterfeld, 2017). Recent work in our lab (Manickam, 2025; https://doi.org/10.1101/2025.06.02.657468) has also shown that TN and TCM may be more inducible by latency reversing agents (LRAs), including HDACis, which may be because effector memory T cells have fewer restrictions to HIV expression, i.e., already have higher levels of expression without LRAs. We therefore believe that the relative increase in the frequency of TCM-like cells suggests that Vpr plays a role in shaping the latent reservoir. We have added some additional discussion of these ideas to the manuscript.**

7) Has differential regulation of different HDACs been examined in T cell subset? This should at least be discussed in greater detail. **The question of differential HDAC activities across T cell subsets is an interesting one, and this has unfortunately not been definitively addressed in the field. Nevertheless, our previous work (Peterson et al 2023, Cell Chemical Biology) has shown that HDACs have a differential effect on the abundance of various memory T cell subsets, as described above, suggesting that the subsets may indeed be differentially regulated by HDACs. We have added some additional speculation about how this may be related to our observations to the manuscript.**

**Part II – Major Issues: Key Experiments Required for Acceptance**
Please use this section to detail the key new experiments or modifications of existing experiments that should be absolutely required to validate study conclusions.

Generally, there should be no more than 3 such required experiments or major modifications for a "Major Revision" recommendation. If more than 3 experiments are necessary to validate the study conclusions, then you are encouraged to recommend "Reject".

Reviewer #1: -

1)Why are the percent positive cells so different between the Thy1.2 and eGFP markers for the flow results? Based on the system, I would have anticipated that percent positive for eGFP would be lower, or at least equivalent, to percent Thy1.2 positive cells. For example, in Figure 1C 63% of the cells are eGFP positive while only 20% are Thy1.2 positive? This changes to 92% eGFP positive and 63% Thy1.2 positive after Vorinostat treatment.

**We apologize for not being clearer in the way the data was presented. The %GFP+ cells is gated within the Thy1.2+ cells, not the overall population of live cells. The reviewer is correct that the %GFP+ cells overall is lower than the %Thy1.2+ cells. We have added arrows to the figures and additional clarification in the text.**

2) The bar graphs throughout are a little misleading for assessing the contribution of Vpr to reactivation of latent reservoirs with vorinostat. In general, Vpr expressing cells activate at ~10-fold lower frequency compared to control cells (ex., Figure 1C control is 18% whereas Vpr cells 1.13%; Figure 3C control is 15.1% while Vpr is 0.62%; Figure 4 control is %6.52 while Vpr is 0.23%). The bar graphs suggest those Vpr infected cells contain brighter eGFP expression, but the flow plots don’t seem to indicate that. Instead, the increase in MFI seems to be due to a smaller denominator, which may not be biologically relevant.
**We would like to apologize again for not being as clear as we hoped. We are not looking at reactivation of viral reservoir in these experiments. Rather, we are looking at relative loss of productively infected cells (GFP+ cells within Thy1.2+ cells) and GFP median fluorescence intensity as a readout for viral gene expression within these productively infected cells as the virus goes into latency/a transcriptionally silent state. Vpr is cytotoxic to infected cells (as shown by Annexin V staining; Fig. S4B), so the percentage of infected cells rapidly declined, but the cells that remained were brighter/had a higher MFI when Vor was also present. All samples with cells infected with HIV-GFP-Vpr had a much lower number of infected cells, but we only saw this MFI increase when cells were treated with Vor. We have added additional clarification in the text/figure legend as well.**

3) The authors should validate that the increase in eGFP MFI in Vpr+vor cells translates to detectable increases in viral protein production compared to control+vor treated cells. This is particularly important given the discrepancy between the flow plots and bar graphs discussed above.
**An important feature of the primary CD4+ T-cell model we use here is that GFP expression is a driven by the endogenous viral promoter and GFP can therefore serve as a clear, accurate proxy for viral protein production. Aside from Tat and Rev, which are difficult to quantify in infected cells, GFP is the only “viral” protein that can be measured since the others have mutations or deletions in their coding regions.**

4) It is odd that several of the Vpr mutants show significantly diminished protein abundance when expressed in cis compared to expression in trans, and that the eGFP MFIs were relatively equivalent. Did the authors confirm that the percent of cells that form latent reservoirs with these new viruses were relatively equivalent compared to wild-type?
**We agree with the reviewer that it is interesting that there was variation in expression levels of the different mutants. We found this variation to be reproducible across multiple experiments. When infecting with our HIV-GFP-Vpr and HIV-GFP viruses, Vpr expression itself is controlled by the viral promoter, which could, in turn, affect how Vpr modulates viral gene expression. The goal of expressing Vpr from an expression vector with a strong promoter was to separate out the effects of Vpr the protein. We were therefore not surprised that these Vpr-expressing lentiviruses resulted in more consistent Vpr protein production. It is possible that some mutants have lower stability, but, as the reviewer mentions, lower Vpr protein levels did not strictly correlate with an abrogation of the effect on GFP MFI, leading us to conclude that the absolute level of Vpr protein in the cell is not a key limiting or determining factor in the experiments. Interestingly, we did observe that p300-binding mutant Vpr also had a faster rate of entry into latency, more similar to HIV-GFP than to wild type Vpr or the other Vpr mutants (S5). We have added some additional discussion to the manuscript regarding this point.**

5) Because several previous studies have suggested that Vpr recruits p300 to the LTR to enhance transcription [PMIDs: 9560267, 12379213, 10505122], I would have anticipated that Vorinostat + p300 inhibition would block the increase in eGFP MFI. The data in the main figures in conjunction with the data presented in Figure S6 suggest this is not the case.

**We agree with the reviewer, and we had also hypothesized that p300i would result in a decreased in the observed effect on GFP MFI. Nevertheless, contrary to our expectations, we did not see this effect with the p300 bromodomain inhibitor, suggesting that recruitment of p300 to chromatin via aceylated histones is not required. However, we did observe a reduction in expression with a p300 histone acetyltransferase (HAT) inhibitor, indicating that the p300 HAT domain is likely more important for the observed increase in viral gene expression in the context of combined Vpr expression and Vor exposure.**

6) Figure 4A indicates a strong increase in CD45A+-CCR7+ cells in Vpr infected cells compared to control cells, but there are significantly fewer Vpr infected cells in the CD45A+-CCR7+ flow plot. Could this increase be due to sampling bias? In other words, if the events were normalized between the plots would the increase observed in the Vpr samples be more comparable to control? For example, the total events in Figure 4B are more similar and the increase in CD45A+-CCR7+ cells in the Vpr infected cells is negligible compared to control cells.

**We thank the reviewer for this point. We think that this is difference is unlikely to be due sampling error. We have now downsampled the data in Flowjo such that the same number of infected (GFP+) cells are gated on in both HIV-GFP-Vpr and HIV-GFP-infected cells, and we see the same effect as with the overall dataset. We have now modified the representative dot plot in Figure 5A to show the results of this downsampling.**

Reviewer #2: No key experiments. 1) Using different HDAC inhibitors would be informative.
**We agree and appreciate the suggestion. We have now added these results to the manuscript and figures (Figure S2A).**

2) Experiments that directly address latency would enhance the significance of findings.
**We have added an experiment in the supplement looking at latency reversal with Vor two weeks post-infection with HIV-GFP or HIV-GFP-Vpr (Figure S2C,D).**

3) In addition, demonstrating Vpr and p300 complexes at the LTR would strengthen the model. **We fully agree with the reviewer, and we devoted a significant amount of time trying to optimize ChIP-PCR or CUT&RUN for low cell numbers, but the cytotoxicity of Vpr that results in 0.5-1% infected cells by week 2, makes this type of experiment unfeasible. We have added a more explicit discussion to this effect. Newer single cell CUT&TAG approaches may be useful to resolve this question in the future, but these methods are highly technically challenging.**

**Part III – Minor Issues: Editorial and Data Presentation Modifications**
Please use this section for editorial suggestions as well as relatively minor modifications of existing data that would enhance clarity.

Reviewer #1: - I’m not sure if it an upload issue or a conversion issue but many of the figures are blurry and the legends are too difficult to read. This is particularly important for trying to interpret the flow cytometry data when comparing percentages across populations.

**We apologize. We will make sure that all figures and legends are clear upon resubmission.**

- Percentage label in Vpr trans mCh experiment in Figure 2D is offset to the right overlapping the other flow plot
**We thank the reviewer and have fixed this issue.**

- S5A labels are shifted

**We thank the reviewer and have adjusted this figure.**

Reviewer #2: Minor comment: Potential indirect effects on HIV transcription should be included on the figure 6 model. Plus, I am not convinced their data support a mechanisms of Vpr+Vor directly influences LTR activity.
**We appreciate the reviewer’s point. We have revised the text to include this possibility.**

Minor comment: ref 21 and 23 are the same?

**We thank the reviewer and have fixed this issue.**
